# Supplementary material for: Copayment mechanism in selected districts of Uganda: Availability, market share and price of quality assured artemisinin-based combination therapies in private drug outlets
Source: PLoS One. 2024 Mar 27;19(3):e0295198. doi: 10.1371/journal.pone.0295198 (PMC10971760; doi:10.1371/journal.pone.0295198)
Supplement: Attachment — Submitted filename: Initial Review Comments .pdf [file pone.0295198.s002.pdf]

The study “Availability, market share and price of quality assured artemisinin-based combination therapies in private drug outlets after over a decade of Copayment mechanism in Uganda” aimed to assess the availability, price, and market share of quality assured artemisinin-based combination therapies (QAACT) in the private drug outlets in low and high malaria transmission settings in Uganda” The authors conducted the study using a cross-sectional design covering and drawing their conclusions based on twenty-eight (28) drug outlets covered.

Overall, it is a useful study as it brings out data that could inform case management but also feeds into the overall ACTs resistance management plan.

General comments

The authors report to have found and included only 28 outlets (drug shops and pharmacies) located in 4 districts covered by the survey. The authors indicate to have used the NDA list of all licensed outlets, conducted a census of all the outlets and enrolled all of them (drug shops and pharmacies) in these districts. Could the authors cross-check if this information is correct? that there are only 28 licensed outlets (pharmacies and drug shops) across the 4 districts?

ACTs procured under the subsidized/copayment mechanism are not distributed to the 4 districts covered by the survey alone but to the entire country of 140 + districts). In my opinion, it be a source of bias to base on four purposely/conveniently picked districts to report and make conclusion on the impact of a decade long program. What do the authors think?

The supply chain system (first line buyers, retailers etc) through which these subsidized/copayment ACTs are distributed entirely relies on market forces, demand creation, etc. Because of these forces and dynamics, there are variations in the market share of these drugs between areas/regions within a country. Eg if you have more distributors moving to same direction you are likely to find more copaid drugs there compared to the rest. The best suitable method to answer the question would therefore entail sampling of outlets in a way that is representative and spread across wide coverage of the country. What do the authors think?

The title of the study appears to be misleading vs the method used and data presented. Copayments/subsidized ACTs in most countries have been supported by donor countries majorly the Global Fund. These grants have time periods within which they expire. After grant expiry, it might take a minimum of 2-3 years to link to the new grant. Therefore, the assumption of 10 years implementing copayment is misleading as there are frequent lapses of non-implementation intervals in between- with the frequent interruption/intervals of non-implementation within the “decade” is the “decade” actually a “decade”

Specific comments

117-118: In all the countries where ACT copayments are being implemented, malaria transmission or endemicity level is not one of the criteria while making decisions around implementing the subsidized/copayments both under the current GF funded grants in Africa and the previously under the AMFm. The major target is to remove barriers to access, availability and affordability particularly in hard to reach and vulnerable populations that are at greatest risk of malaria infections regardless of transmission/endemicity level. The convenient selection of districts from low and high transmission doesn’t seem to add value in view of the key goals of copayments.

202-203: Survey covered 28 outlets- small size samples are characterized by wide confidence intervals. It is not clear whether the P-values obtained are not due to small size/numbers. The

confidence intervals are not shown throughout the report. What do the authors think about the strength of this evidence in view of such important intervention?

Table 1: ACT Copayments are designed to achieve cost /price reduction and access, availability- it would be more informative if the table was stratified to bring out the key copayment aspects

110-111: There are several programmatic reports (unpublished) that have evaluated the effect of ACT copayment. It would be great to access and see what these reports say vs this study

117-118: malaria epidemiology is highly dynamic and transmission is highly heterogenous and this complicates epidemiological stratification into high, Moderate and low transmission based on parasite prevalence/slide positivity rates alone. Areas that were originally high are now low and vice versa and this phenomenon is happening in most countries in Africa. Accurate Epidemiological stratification is based on a composite factor that combines EIRs, parasite rates & positivity rates, etc. what parameters were based arrive at the transmission levels are reported? Is there data on the EIRs for these areas indicated?

122-126: were there discrepancies between the NDA list and the physical census?

139: HAI Write in full time first

Line 354: While the conclusion is strong, the evidence /data and the methods used appear sub-optimal to support it (for the reasons elaborated above)

1. The selection was purposively and conveniently selected- which could have introduced bias. There was no attempt to address this at analysis either through regression to adjust for it or by any other means
2. A sample of 28 outlets is certainly too small to draw conclusions on a decade long intervention that covers the entire country
3. Conducting a simple cross-sectional survey at the end of an important project such as this, would assume a decade long, contentious uninterrupted implementation of ACT copayment/subsided ACT project which would be rather misleading. There are about three incidents of 2-3 year periods of interrupted non-implementation. A cross sectional study certainly masks important information within the decade.
4. The study looked at outlets in high and low transmission, a criterion that is not very useful for the ACTS Copayments (access, cost, hard to reach, etc)
5. The epidemiological criteria for stratifying districts into low and high was not was not indicated
