# Supplementary material for: Copayment mechanism in selected districts of Uganda: Availability, market share and price of quality assured artemisinin-based combination therapies in private drug outlets
Source: PLoS One. 2024 Mar 27;19(3):e0295198. doi: 10.1371/journal.pone.0295198 (PMC10971760; doi:10.1371/journal.pone.0295198)
Supplement: Attachment — Submitted filename: RESPONSE TO REVIEWER.pdf [file pone.0295198.s003.pdf]

RESPONSE TO REVIEWER'S COMMENTS PONE-D-23-37429

We are grateful for the comments from the reviewers of our manuscript. The comments are insightful and responding to them have helped improve the manuscript. Find below a summary of our responses to the comments raised on the manuscript by the reviewers.

Comment #1: The study "Availability, market share and price of quality assured artemisinin-based combination therapies in private drug outlets after over a decade of Copayment mechanism in Uganda" aimed to assess the availability, price, and market share of quality assured artemisinin-based combination therapies (QAACT) in the private drug outlets in low and high malaria transmission settings in Uganda" The authors conducted the study using a cross-sectional design covering and drawing their conclusions based on twenty-eight (28) drug outlets covered. Overall, it is a useful study as it brings out data that could inform case management but also feeds into the overall ACTs resistance management plan.

Response: Thanks for the appreciation.

General comments

Comment #2: The authors report to have found and included only 28 outlets (drug shops and pharmacies) located in 4 districts covered by the survey. The authors indicate to have used the NDA list of all licensed outlets, conducted a census of all the outlets and enrolled all of them (drug shops and pharmacies) in these districts. Could the authors cross-check if this information is correct? that there are only 28 licensed outlets (pharmacies and drug shops) across the 4 districts?

Response: Thanks for the comments, it is not true that there are only 28 drug outlets in the four districts. As per the drug outlet register available at https://www.nda.or.ug/licensed-outlets/ (Accessed 30th November 2023) there are a total of 122 pharmacies in the four districts (Mbarara, 88; Kabale, 16; Tororo, 16 and Apac, 2). Mbarara was recently divided into a city and district, and we collected data in the city not the district. As per the website, Mbarara city has 45 pharmacies. From the census and review of drug outlets listed in the NDA register, each of the available drug outlet in the survey districts were considered for inclusion in the survey. The drug outlet which reported stocking antimalarial agents were noted and the batches of different individual antimalarial agents were used to determine if the agent would be included in the survey. Data on the antimalarial agent was collected whenever a batch that has not been encountered before was found in the drug outlet. Data on each batch was collected once in a total of 28 different private drug outlets. This has been clarified in the revised manuscript.

Comment #3: ACTs procured under the subsidized/copayment mechanism are not distributed to the 4 districts covered by the survey alone but to the entire country of 140 + districts). In my opinion, it be a source of bias to base on four purposely/conveniently picked districts to report and make conclusion on the impact of a decade long program. What do the authors think?

Response: Thanks for the comment, true the subsidized ACTs are not distributed in only the 4 districts included in this survey. The WHO/HAI recommends a total of 30 drug outlets (public and private) from six survey areas for medicine availability and price studies (5 drug outlets per survey area). In Uganda, medicines are supplied free of charge to patients in public health facilities and thus we did not include public facilities in the survey areas. We had a total of 4 survey areas and 28 drug outlets (22 pharmacies and 6 drug shops). We included a total of 144 antimalarial agents in the survey. The four districts were purposely selected to represent the malaria transmission patterns of the country. Both low and high malaria transmission settings are included in this study which is more likely to represent the true picture of malaria situation in the country. Apac and Tororo districts represent are the districts with the highest malaria transmission in the country. Kabale and Mbarara districts are the districts with lowest malaria transmission in the country. While the districts were purposely selected, all the available drug outlets in each district were visited and considered for inclusion in the survey reducing the risk of bias. Our findings are thus a true reflection of situation of subsidized ACTs in the country.

Comment #4: The supply chain system (first line buyers, retailers etc) through which these subsidized/copayment ACTs are distributed entirely relies on market forces, demand creation, etc. Because of these forces and dynamics, there are variations in the market share of these drugs between areas/regions within a country. Eg if you have more distributors moving to same direction you are likely to find more copaid drugs there compared to the rest. The best suitable method to answer the question would therefore entail sampling of outlets in a way that is representative and spread across wide coverage of the country. What do the authors think?

Response: Thanks for the comment, true the distribution of the subsidized ACTs is likely to follow market forces as suggested by the reviewer. In our study we assessed the subsidized ACTs in both low and high malaria transmission settings which represent settings of potentially high demand due to high malaria transmission and low demand due to low malaria transmission. We agree with the reviewer, and this was factored in our study with the inclusion of areas of high and low malaria transmission. However, from the findings of our study there was no significant difference in the distribution of QAACT by malaria transmission setting ($p=0.35$) which is in line with the reviewer's comment. This is highlighted in the revised manuscript.

Comment #5: The title of the study appears to be misleading vs the method used and data presented. Copayments/subsidized ACTs in most countries have been supported by donor countries majorly the Global Fund. These grants have time periods within which they expire. After grant expiry, it might take a minimum of 2-3 years to link to the new grant. Therefore, the assumption of 10 years implementing copayment is misleading as there are frequent lapses of non-implementation intervals in between- with the frequent interruption/intervals of non-implementation within the "decade" is the "decade" actually a "decade"

Response: The Affordable Medicines Facility for malaria (AMFm) pilot program was launched in 2010 consisting of nine pilots in eight countries: Cambodia, Ghana, Kenya, Madagascar, Niger, Nigeria, Uganda, Tanzania mainland and Zanzibar (The Global Fund to

Fight AIDS, Tuberculosis and Malaria, 2008; ACTwatch et al., 2017). The pilot phase of program was from 2010 to 2011. After the expiry of the pilot phase, the AMFm transitioned to the private sector copayment mechanism (CPM) in 2013 in six countries: Ghana, Kenya, Madagascar, Nigeria, Tanzania, and Uganda. The co-payment mechanism has been implemented in Uganda since 2013 albeit with various challenges. Although the program has was launched in 2013, its implementation has been faced with various challenges which have caused interruptions to the program. We have published separately demonstrating the various challenges this program has faced in the country. We have removed the word 'after a decade' in the title of our study as guided by the reviewer.

Specific comments

Comment #6: 117-118: In all the countries where ACT copayments are being implemented, malaria transmission or endemicity level is not one of the criteria while making decisions around implementing the subsidized/copayments both under the current GF funded grants in Africa and the previously under the AMFm. The major target is to remove barriers to access, availability and affordability particularly in hard to reach and vulnerable populations that are at greatest risk of malaria infections regardless of transmission/endemicity level. The convenient selection of districts from low and high transmission doesn't seem to add value in view of the key goals of copayments.

Response: The prevalence of malaria is not uniform across Uganda which potentially creates variation in demand for the antimalarial agents in the country. In our study we ensured that the low and high malaria transmission (burden) settings are represented. Since our study was focused on the private sector, the design helped ensure representation of the settings of potentially low and high demand for the subsidized ACTs. However, from the findings of our study there was no significant difference in the distribution of QAACT by malaria transmission setting (p=0.35*)* which is in line with the reviewer's comment. This is highlighted in the revised manuscript.

Comment #7: 202-203: Survey covered 28 outlets- small size samples are characterized by wide confidence intervals. It is not clear whether the P-values obtained are not due to small size/numbers. The confidence intervals are not shown throughout the report. What do the authors think about the strength of this evidence in view of such important intervention?

Response: The WHO/HAI recommends a total of 30 drug outlets (public and private) from six survey areas for medicine availability and price studies (5 drug outlets per survey area). In Uganda, medicines are supplied free of charge to patients in public health facilities and thus we did not include public facilities. In all the districts, we sampled more than 5 drug outlets. We had a total of 4 survey areas and 28 drug outlets (22 pharmacies and 6 drug shops). We included a total of 144 antimalarial agents in the survey. We have also included confidence intervals to our estimates indicating good precision in our estimates as there are no wide confidence intervals.

<u>*Reference:*</u>

WHO/HAI [Health action international/World Health Organization] Medicine prices: a new approach to measurement. 2003 Ed. Geneva, World Health Organization and Health Action International, 2003.

Comment #8: Table 1: ACT Copayments are designed to achieve cost /price reduction and access, availability- it would be more informative if the table was stratified to bring out the key copayment aspects.

Response: Thanks for the comment. Our results cover availability and price which are the key aspects of the copayment mechanism. The results are provided under the following sections in the manuscript; *'Price of antimalarial agents in drug outlets in Uganda'* and *'Price mark-ups on quality assured artemisinin-based combination therapies (QAACTs) in drug outlets in Uganda'*

Comment #9: 110-111: There are several programmatic reports (unpublished) that have evaluated the effect of ACT copayment. It would be great to access and see what these reports say vs this study

Response: Thanks for the comment, we have reviewed both published and unpublished work in writing and revising the manuscript.

Comment #10: 117-118: malaria epidemiology is highly dynamic, and transmission is highly heterogenous, and this complicates epidemiological stratification into high, Moderate and low transmission based on parasite prevalence/slide positivity rates alone. Areas that were originally high are now low and vice versa and this phenomenon is happening in most countries in Africa. Accurate Epidemiological stratification is based on a composite factor that combines EIRs, parasite rates & positivity rates, etc. what parameters were based arrive at the transmission levels are reported? Is there data on the EIRs for these areas indicated?

Response: We used the Ministry of Health stratification of malaria transmission settings across the country to identify areas categorized as having high and low malaria transmission. Tororo and Apac districts are categorized as having high malaria transmission and Mbarara and Kabale are low malaria transmission areas. This information has been incorporated in the revised manuscript.

Comment #11: 122-126: were there discrepancies between the NDA list and the physical census?

Response: Yes, most of the drug outlets listed on the NDA website are not found on ground.

Comment #12: 139: HAI Write in full time first.

Response: Thanks for the comment, this was already provided in the abstract.

HAI: Health Action International. This is provided in the revised manuscript.

Comment #13: Line 354: While the conclusion is strong, the evidence /data and the methods used appear sub-optimal to support it (for the reasons elaborated above)

Response: Thanks, we have addressed the comments raised on the manuscript and believe that our findings support the conclusion and reflects the situation of copayment mechanism in the country.

Comment #14: 1. The selection was purposively and conveniently selected- which could have introduced bias. There was no attempt to address this at analysis either through regression to adjust for it or by any other means

Response: Thanks for the comment, in the revised manuscript we have conducted regression analysis and the results have been incorporated in the revised manuscript.

Comment #15: 2. A sample of 28 outlets is certainly too small to draw conclusions on a decade long intervention that covers the entire country.

Response: Thanks for the comment. The WHO/HAI recommends a total of 30 drug outlets (public and private) from six survey areas for medicine availability and price studies (5 drug outlets per survey area). In Uganda, medicines are supplied free of charge to patients in public health facilities and thus we did not include public facilities in the survey areas. We had a total of 4 survey areas and 28 drug outlets (22 pharmacies and 6 drug shops). We included a total of 144 antimalarial agents in the survey. We have also included confidence intervals to our estimates indicating good precision in our estimates. The study found 13.8% (20/144: 95%CI: 9.1, 20.6) availability of quality assured artemisinin-based combination therapy (QAACT) in private drug outlets. We also conducted regression analysis and results are provided in the revised manuscript. The factors associated with availability of quality assured artemisinin-based combination therapy was assessed using modified Poisson regression at bivariate level to establish the crude relationship between the predictors and the outcome variable, and multivariate levels to assess the adjusted relationship between the predictors and the outcome variables. Factors with P-value of 0.2 and below at bivariate analysis were included in the multivariable regression analysis. Statistical significance was determined at 5% level of significance. The results of the regression analysis are presented, and all have narrow confidence intervals.

At bivariate regression analysis, the factors associated with availability of QAACT in drug outlets include pharmacy (cPR: 0.48; 95%CI: 0.2, 1.1; *p*=0.07), low malaria transmission (cPR:1.83; 95%CI: 0.11, 0.5; *p*=0.35), and dispensing price > 3000UGX (USD=0.83) (cPR: 0.23; 95%CI: 0.11, 0.5; *p*=<0.000).
Multivariable regression analysis, the predictors of availability of QAACT in drug outlets include pharmacy (aPR:0.4; 95%CI: 0.2, 0.9) and having dispensing price more than 3000UGX (USD 0.83) (aPR: 0.4, 95%CI: 0.1, 0.51) (Table 3). The ACTs that were priced more than USD 0.83 were 77% less likely to be quality assured. The ACTs in pharmacies were 52% less likely to be quality assured.

Table 3: Predictors of availability of quality assured ACTs in drug outlets

Characteristic	Description	cPR (95%CI)	aPR (95%CI)
Drug outlet	Drug shop	1.0	1.0
	Pharmacy	0.48 (0.2, 1.1)	0.4 (0.2, 0.9)
Malaria transmission setting	High	1.0	-
	Low	1.83 (0.51, 6.51)	-
Price category	<3000 UGX (USD 0.83)	1.0	1.0
	>3000 UGX (USD 0.83)	0.23 (0.11, 0.5)	0.23 (0.1, 0.51)

cPR: crude prevalence ration, aPR: adjusted prevalence ration, CI: Confidence interval, UGX: Ugandan Shillings

Comment #16: 3. Conducting a simple cross-sectional survey at the end of an important project such as this, would assume a decade long, contentious uninterrupted implementation of ACT copayment/subsided ACT project which would be rather misleading. There are about three incidents of 2-3 year periods of interrupted non-implementation. A cross sectional study certainly masks important information within the decade.

Response: Thanks for the comment, information on the interruptions to the implementation of copayment mechanism in Uganda is not readily available. We also followed the standard criteria by WHO/HAI for assessment of availability and price of medicines. However, we have adjusted the title of the manuscript to remove the word 'decade'.

Comment #17: 4. The study looked at outlets in high and low transmission, a criterion that is not very useful for the ACTS Copayments (access, cost, hard to reach, etc)

Response: The use of low and high malaria transmission setting was to ensure representation of areas of potentially varied demand for antimalarial agents. This is especially the case due to the influence of market forces of demand and supply in the private sector. However, from the findings of our study there was no significant difference in the distribution of QAACT by malaria transmission setting (p=0.35*)* which is in line with the reviewer's comment. This is highlighted in the revised manuscript.

Comment #18: 5. The epidemiological criteria for stratifying districts into low and high was not not indicated.

Response: Thanks for the comment, this has been clarified in the revised manuscript. We used the ministry of health categorization of malaria transmission settings for identification of areas of high and low malaria transmission in the country. From the Ministry of health classification, we selected Tororo and Apac for high; then Mbarara and Kabale for low malaria transmission setting.

Comment #19: 6. overall, the methods and the data generated, and the evidence reported do not adequately support the conclusion for such an important important intervention.

Response: Thanks for the comment, we followed the standard criteria by WHO/HAI for assessment of availability and price of medicines. In the survey areas (districts) all the drug outlets were considered for inclusion. Additionally, data was collected in settings of the country that represent areas with potentially low and high demand for subsidized ACTs. Addressing all the corrections as requested by the reviewers has further improved the manuscript.
