# Supplementary material for: Copayment mechanism in selected districts of Uganda: Availability, market share and price of quality assured artemisinin-based combination therapies in private drug outlets
Source: PLoS One. 2024 Mar 27;19(3):e0295198. doi: 10.1371/journal.pone.0295198 (PMC10971760; doi:10.1371/journal.pone.0295198)
Supplement: Attachment — Submitted filename: RESPONSE TO REVIEWER_PONE-D-23-37429R1_1March24.doc [file pone.0295198.s004.doc]

**RESPONSE TO REVIEWER’S COMMENTS OF MANUSCRIPT** PONE-D-23-37429R1

Title: Copayment mechanism in Uganda: Availability, market share and price of quality assured artemisinin-based combination therapies in private drug outlets

**Editor’s comment:** The study aims to assess the availability, price, and market share of quality assured artemisinin-based combination therapies (QAACT) in the private drug outlets in low and high malaria transmission settings in Uganda” The authors conducted the study using a cross-sectional design covering and drawing their conclusions based on twenty-eight (28) drug outlets covered.
Overall, it is a useful study as it brings out data that could inform case management but also feeds into the overall ACTs resistance management plan.

Having read the concerns raised by the reviewers who are both subject matter experts at the WHO global malaria program and in my own view, there are some issues of methodological nature which should further improve the paper if addressed.

**Response:** Thanks for the feedback, we have addressed the additional comments from both the editor and the reviewer’s which has helped further improve the manuscript.

**Editor’s comment #1:** the ACTs procured under the subsidized/copayment mechanism are not distributed to the 4 districts covered by the survey alone but to the entire country of 140 + districts). In my opinion, it a big source of bias to base on four purposely/conveniently picked districts to report and make conclusion on the impact of a decade long program. This has also been raised by expert reviewer 2. It would generally be a good and informative study if reported findings from the 4 four the four study districts including a disclaimer that these findings do not reflect the effects of copayments in Uganda but in the study districts.

**Response:** This has been noted, and its true the subsidized ACTs are distributed to the entire country. We have adjusted the write up in the manuscript to reflect this and included in the limitation and conclusion of the study.

**Editor’s comments #2:** As raised earlier The supply chain system (first line buyers, retailers etc) through which these subsidized/copayment ACTs are distributed entirely relies on market forces, demand creation, etc. Because of these forces and dynamics, there are variations in the market share of these drugs between areas/regions within a country. Eg if you have more distributors moving to same direction you are likely to find more copaid drugs there compared to the rest. The best suitable method to answer the question would therefore entail sampling of outlets in a way that is representative and spread across wide coverage of the country. In their response, the authors mentioned that they samples from the various epidemiological endemicity but that does not address the risk of selection of bias due to an up-hazard distribution system that may cause over supply of co-paid drugs in some areas compared to the others. The up-hazard distribution and selective and non-probability convenient selection of districts makes generalization of this study difficult.

**Response:** Thanks for the comment, we have adjusted the title and conclusion to limit the findings to only the four (4) districts where data was collected.

**Editor’s comments #3:** Reviewer 2 raises a very important aspect about the changing malaria epidemiology in Africa (Uganda inclusive)- malaria epidemiology is highly dynamic, and transmission is highly heterogenous, and this complicates epidemiological stratification into high, Moderate and low transmission merely based on parasite prevalence/slide positivity rates alone. Areas that were originally high are now low and vice versa and this phenomenon is happening in most countries in Africa. Accurate Epidemiological stratification is based on a composite factor that combines entomological inoculation rates (EIRs), parasite rates & positivity rates, etc. As raised by reviiwer 2 it nt clear how the decision stratify into high and low transmission was reached- raising questions on whether study areas such as Tororo and Apac are correctly epidemiologically placed. what parameters were based arrive at the transmission levels.

**Response:** We followed the Ministry of Health stratification of the malaria transmission intensity in the country. According to a publication by WHO in 2023 Uganda has the world’s highest malaria incidence rate of 478 cases per 1,000 population per year. It is also the leading cause of sickness and death in Uganda and is responsible for up to 40 percent of all outpatient visits, 25 percent of hospital admissions and 14 percent of all hospital deaths. The malaria death rate in Uganda is estimated to be between 70,000- 100,000 deaths per year (WHO malaria country report, 2023). Malaria transmission is stratified into four levels based on malaria cases, ‘high’ (> 499 cases per 1000 population/year), ‘moderate’ (200 to 499 cases per 1000 population per year), ‘low’ level 2 (50 to 199 cases per 1000 population per year), ‘very low’ level 1 (1-49 cases per 1000 population per year) (Zalwango et al., 2022).

The malaria test positivity rate (TPR) in Tororo district decreased from 60% (2012 to 2013) to 31% during final period of Actellic indoor residual spraying (IRS) and LLIN (June 2016 to December 2019) (Mpimbaza et al., 2020; Nankabirwa et al., 2022). Although there has been remarkable decrease in malaria prevalence in Tororo and Apac districts, these gains remain fragile due to inconsistency in implementation of the malaria vector control measures (Nankabirwa et al., 2022). Previous studies in Uganda (Esptein et al., 2023; Epstein et al., 2022) reported a resurgence in malaria burden to pre-IRS levels despite sustained IRS across sentinel surveillance sites. Therefore, despite implementation of various control interventions, malaria remains a public health concern in historically high burden districts in North-Eastern Uganda like Apac and Tororo districts where the current study was conducted.

Uganda Bureau of Statistics (UBOS) and ICF. 2018. Uganda Demographic and Health Survey 2016. Kampala, Uganda and Rockville, Maryland, USA: UBOS and ICF.

Nankabirwa JI, Bousema T, Blanken SL, Rek J, Arinaitwe E, Greenhouse B, et al. (2022) Measures of malaria transmission, infection, and disease in an area bordering two districts with and without sustained indoor residual spraying of insecticide in Uganda. PLoS ONE 17(12): e0279464

Mpimbaza, A., Sserwanga, A., Rutazaana, D. *et al.* Changing malaria fever test positivity among paediatric admissions to Tororo district hospital, Uganda 2012–2019. *Malar J* 19, 416 (2020).

Epstein A, Maiteki-Sebuguzi C, Namuganga JF, Nankabirwa JI, Gonahasa S, Opigo J, Staedke SG, Rutazaana D, Arinaitwe E, Kamya MR, Bhatt S, Rodríguez-Barraquer I, Greenhouse B, Donnelly MJ, Dorsey G. Resurgence of malaria in Uganda despite sustained indoor residual spraying and repeated long lasting insecticidal net distributions. PLOS Glob Public Health. 2022 Sep 7;2(9):e0000676.

Epstein A, Namuganga JF, Nabende I, et al. Mapping malaria incidence using routine health facility surveillance data in Uganda. BMJ Global Health 2023;8:e011137

**Editor’s comments #4:** Overall, this is a very useful that is publishable- however the findings should be tailored to the 4 study districts. Like wise, conclusions can only be true for the study areas (would be better to retain the current title but add something like "..........; a case of four districts in Uganda"

**Response:** Thanks for the comment, the title has been revised to reflect the fact that the study was conducted in four (4) districts.

**Reviewer’s comment #1:** General:
Well written paper which gives a context for the improvement to access to quality assured ACTs across all sectors of the health system, particularly the private sector. However, a few areas (indicated below) would need minor adjustments to give the reader a true context of the situation and thus help in addressing the challenges this study has identified.

**Response:** Thanks for the comment and have addressed this in the revised manuscript.

**Reviewer’s comment #2**. Though Co-payment mechanism is over 10 years in Uganda, the context has changed overtime. It would be helpful to give a summary of the Co-payment mechanism as at the time of the study, e.g. which antimalarial medicines in the national guidelines is a beneficiary of the scheme. A sentence in the introduction reads "The approved quality assured ACTs in Uganda include Artemether/lumefantrine, Artesunate/Amodiaquine, Dihydroartemisinin/piperaquine, and Artesunate/Pyronaridine", is not factual. Please clarify, is there dihydroartemisin/piperaquine and artesunate/pyronaridine with the "Green leaf" logo available. So though it is correct that all the medicines indicated in your statement are approved for use in Uganda, but not all of them benefit from co-payment scheme presently. Please clarify and modify the paper and conclusion accordingly.

|  | **Response:** As indicated in the manuscript, the approved quality assured ACTs are as indicated in the figure.  <https://onehealthtrust.org/publications/archive/affordable-medicines-facility-malaria-ugandas-perspective/> |
| --- | --- |

**Reviewer’s comment #3**. Price of antimalarial agents in drug outlets in Uganda: - Some of the prices stated in the text does not match what is given in Table 2. Cross-check for consistency.

**Response:** Thanks for the observation, this has been rectified in Table 2 of the revised manuscript.

**Reviewer’s comment #4**. Price mark-ups on quality assured artemisinin-based combination therapies (QAACTs) in drug outlets in Uganda:-
What factors determine the final price? What is the buying price of the QAACT from first line buyers? Are the first line buyers maintaining the recommended selling prices to the pharmacies and drug shops?

**Response:** We did not assess the buying price of first line buyers. However, in a separate qualitative study (Manuscript under review) we explored and reported on the determinants of final QAACT price in the private drug outlets in selected districts in Uganda. In this study we focused on access to QAACT by the malaria patients in private drug outlets. We also report on concordance between dispensing price of QAACT in private drug outlets and the recommended retail prices as per the Ministry of Health.

**Reviewer’s comment #5:** The conclusion that – “The results of our study indicate low prevalence of QAACTs in private drug outlets despite the implementation of private sector copayment mechanism in Uganda” is devoid of any contextual factors as to the finding. If these were collected as part of the study, it would enhance the strength and usefulness of the study results if they can be added to the paper.

**Response:** We collected contextual factors and performed multivariable logistic regression and the results are reported in the manuscript.

**Reviewer’s comment #6:** This paper addresses a topical issue on access to ACT therapy in Uganda. I however have some comments that need to be addressed.

**Response:** We are grateful for the comments, addressing these comments have improved our manuscript.

**Reviewer’s comment #7:** In the background it is stated that that malaria accounts for 30-50% OPD attendances and 15-20 Hospital admissions. These data are almost 3 decades old. Is it really true that with recent interventions these statics have not changed?Secondly with IRS and LLINS implementation, is Apac and Tororo still ranked as high transmission areas. Please provide recent evidence to justify this stratification. This paper also misses out major papers on this subject in Uganda e.g Talisuna et al 2012, Gunther et al 2014

**Response:** Malaria contributing to 30-50% of OPD attendances and 15-20 Hospital admissions is within the range of the current observed malaria burden in the country. According to a publication by WHO in 2023 Uganda has the world’s highest malaria incidence rate of 478 cases per 1,000 population per year. It is also the leading cause of sickness and death in Uganda and is responsible for up to 40 percent of all outpatient visits, 25 percent of hospital admissions and 14 percent of all hospital deaths (WHO, malaria report, 2023; MoH, 2024).

The malaria death rate in Uganda is estimated to be between 70,000 and 100,000 deaths per year (WHO malaria country report, 2023). Malaria transmission which is stratified into four levels based on malaria cases, ‘high’ (> 499 cases per 1000 population/year), ‘moderate’ (200 to 499 cases per 1000 population per year), ‘low’ level 2 (50 to 199 cases per 1000 population per year), ‘very low’ level 1 (1-49 cases per 1000 population per year) (Zalwango et al., 2022).

The malaria test positivity rate (TPR) in Tororo district decreased from 60% (2012 to 2013) to 31% during final period of Actellic indoor residual spraying (IRS) and LLIN (June 2016 to December 2019) (Mpimbaza et al., 2020; Nankabirwa et al., 2022). Although there has been remarkable decrease in malaria prevalence in Tororo and Apac districts, these gains remain fragile due to inconsistency in implementation of the malaria vector control measures (Nankabirwa et al., 2022). Previous studies in Uganda (Esptein et al., 2023; Epstein et al., 2022; Talisuna et al., 2012) reported a resurgence in malaria burden to pre-IRS levels despite sustained IRS across sentinel surveillance sites. Therefore, despite implementation of various control interventions, malaria remains a public health concern in historically high burden districts in North-Eastern Uganda like Apac and Tororo districts where the current study was conducted. Based on the current evidence on malaria burden in the study districts, we have revised the categorization of Apac and Tororo as having moderate-to-high malaria transmission (Talisuna et al., 2012; Esptein et al., 2022).

Talisuna A, Adibaku S, Dorsey G, Kamya MR, Rosenthal PJ. Malaria in Uganda: challenges to control on the long road to elimination. II. The path forward. Acta Trop. 2012 Mar;121(3):196-201.

Zalwango, M.G., Zalwango, J.F., Kadobera, D. *et al.* Evaluation of malaria outbreak detection methods, Uganda, 2022. *Malar J* **23**, 18 (2024).

Nankabirwa JI, Bousema T, Blanken SL, Rek J, Arinaitwe E, Greenhouse B, et al. (2022) Measures of malaria transmission, infection, and disease in an area bordering two districts with and without sustained indoor residual spraying of insecticide in Uganda. PLoS ONE 17(12): e0279464

Mpimbaza, A., Sserwanga, A., Rutazaana, D. *et al.* Changing malaria fever test positivity among paediatric admissions to Tororo district hospital, Uganda 2012–2019. *Malar J* 19, 416 (2020).

Epstein A, Maiteki-Sebuguzi C, Namuganga JF, Nankabirwa JI, Gonahasa S, Opigo J, Staedke SG, Rutazaana D, Arinaitwe E, Kamya MR, Bhatt S, Rodríguez-Barraquer I, Greenhouse B, Donnelly MJ, Dorsey G. Resurgence of malaria in Uganda despite sustained indoor residual spraying and repeated long lasting insecticidal net distributions. PLOS Glob Public Health. 2022 Sep 7;2(9):e0000676.

Uganda Bureau of Statistics (UBOS) and ICF. 2018. Uganda Demographic and Health Survey 2016. Kampala, Uganda and Rockville, Maryland, USA: UBOS and ICF.

**Reviewer’s comment #8**: A second major limitation of this study is the sample size estimation of the number of the districts selected compared to the total number of districts in Uganda. As of December 2023, Uganda had close to 135 districts, so to conduct a study in 4 districts and then generalize the findings to 135 districts is Epidemiological injustice

**Response:** Thanks for the comment, the title of the study, conclusion and overall write-up has been adjusted to reflect the fact that data was collected from only four districts in the country. However, the four districts cover diverse malaria transmission settings in the country.

**Reviewer’s comment #9**. The title of the study should be "Co-payment mechanism in Select District in Uganda: Availability, market share and price of quality assured artemisinin-based combination therapies in private drug outlets,

**Response:** Thanks, this has been incorporated in the revised manuscript.

**Reviewer’s comment #10**. The Generalised conclusion both in the abstract and the main text needs to b revised what was studies here does surely not represent Uganda as close to 30 districts should have been sample

**Response:** Thanks for the comment, we have restricted the conclusion in the revised manuscript to the four (4) districts from which the data was collected.

**Reviewer’s comment #11:** Data us plural so the paper should be revised to reflect that " Data were analyzed using instead of data was analyzed using.

**Response:** Thanks, this has been corrected in the revised manuscript.
